# Outpatient care for adolescents’ and young adults’ mental health: promoting self- and others’ understanding through a metacognitive interpersonal therapy-informed psychological intervention

**DOI:** 10.3389/fpsyt.2023.1221158

**Published:** 2023-11-02

**Authors:** Elisa Marconi, Laura Monti, Giulia Fredda, Georgios D. Kotzalidis, Delfina Janiri, Valentina Zani, Debora Vitaletti, Maria Velia Simone, Simone Piciollo, Federica Moriconi, Emanuela Di Pietro, Raffaele Popolo, Giancarlo Dimaggio, Chiara Veredice, Gabriele Sani, Daniela Pia Rosaria Chieffo

**Affiliations:** ^1^Clinical Psychology Unit, Fondazione Policlinico Universitario Agostino Gemelli IRCCS, Rome, Italy; ^2^Department of Psychiatry, Department of Neuroscience, Head, Neck and Thorax, Fondazione Policlinico Universitario Agostino Gemelli IRCCS, Rome, Italy; ^3^NESMOS Department (Neurosciences, Mental Health, and Sensory Organs), University of Rome “La Sapienza”, Rome, Italy; ^4^Catholic University of the Sacred Heart–Rome, Rome, Italy; ^5^Center for Metacognitive Interpersonal Therapy, Turin, Italy; ^6^Center for Metacognitive Interpersonal Therapy, Rome, Italy; ^7^Department of Mental Health, Rome, Italy; ^8^Pediatric Neuropsychiatry Unit, Fondazione Policlinico Universitario Agostino Gemelli IRCCS, Rome, Italy; ^9^Institute of Psychiatry, Department of Neuroscience, Catholic University of the Sacred Heart–Rome, Rome, Italy; ^10^Department of Life Sciences and Public Health Department, Catholic University of Sacred Heart, Rome, Italy

**Keywords:** metacognitive interpersonal therapy, psychotherapy, adolescence, treatment adherence, general psychopathology, drop-out rate, functioning

## Abstract

**Introduction:**

Psychological distress may result in impairment and difficulty understanding oneself and others. Thus, addressing metacognitive issues in psychotherapy may improve psychopathology in adolescents and young adults (AYAs). We aimed to compare metacognitive interpersonal therapy (MIT)-informed psychotherapy with other treatment-as-usual (TAU) therapies.

**Methods:**

We administered the Global Assessment of Functioning (GAF) scale, the Clinical Global Impressions–Severity (CGI-S) scale, and the Brief Psychiatric Rating Scale (BPRS) at baseline (BL) and at treatment termination (the endpoint was at 6 months and any last results obtained before that term were carried forward in analyzes). Patients received concomitant psychiatric and psychological treatment.

**Results:**

Sixty AYAs were involved in the study. There was a significant reduction in symptomatology after the intervention. Twelve patients (17%) dropped out; treatment adherence was 83%. In the MIT group, 2 patients dropped out (11%), and in the TAU group, 9 patients dropped out (19%). All scales showed a significant reduction in symptoms between baseline (BL) and the 6-month endpoint: GAF (*χ*^2^ = 6.61, *p* < 0.001), BPRS (*χ*^2^ = 6.77, *p* < 0.001), and CGI (*χ*^2^ = 7.20, *p* < 0.001). There was a greater efficacy for the MIT group in terms of symptom reduction on the BPRS (*t* = 2.31; *p* < 0.05).

**Conclusion:**

The study confirmed the efficacy of early and integrated care in adolescence and suggested greater symptom reduction for a psychotherapeutic intervention focused on stimulating mentalization skills. The study indicates the usefulness of this type of approach in the treatment of adolescent psychopathology. Due to the small sample size, the results need replication.

## 1. Introduction

The World Health Organization (WHO) (1) reported that 10–20% of children and adolescents had mental health problems prior to the COVID-19 outbreak. The onset of about half of mental disorders occurs within 14 years of age, with three-fourths having their onset before age 18 (2, 3). Generalized anxiety disorder and depression are the most common disorders (4), and their prevalence among young people has risen in the last 25 years (5). Depression and anxiety impact adolescent development negatively, including lower academic performance, school dropout, strained social relationships, increased risk of substance abuse, self-harm, and suicide (6–9).

The COVID-19 emergency caused a health alert and sudden changes in daily life and mental disorders (10–12). In children, depression and anxiety were among the most common mental disorders (13, 14). Young people suffer behavioral and emotional changes, such as sleep problems, phobias, increased drug and alcohol use, isolation, loss of interest, and irritability (15). Although in this study we will not focus on the effects of the pandemic on adolescent and young adult patients’ (AYA) treatment, the pandemic affected patient recruitment and service access. Several studies aimed to identify effective interventions suiting adolescents’ needs during the pandemic (16–21).

Greater accuracy and definition of care pathways would possibly combine emerging evidence with proven pharmacological (22, 23), psychotherapeutic (23–25), and combined interventions (26, 27).

Cognitive behavior therapy (CBT) is effective in treating anxiety and depression (28). However, outcomes are not yet entirely satisfactory, mainly due to high dropout and insufficient remission rates. Studies reported low response and premature psychotherapy termination, especially for adolescents with moderate to severe anxiety and depression. Concerning dropout, one study of 406 patients reported it to be 37% (29), a meta-analysis reported 23% (30), and other studies reported lower rates [12.2% (31)–13.3% (32)].

Another issue is remission. A meta-analysis of CBT in childhood and adolescence (28) showed remission to occur in only 53.2% of adolescents with depressive and 50.7% of adolescents with anxiety disorders. Against this background, current protocols clearly need improvement.

Since the capacity to reflect on mental states and use psychological knowledge for purposeful problem-solving is reduced in many patients, it is reasonable to consider it a relevant treatment target (33). One of the underlying reasons for adopting the metacognitive interpersonal therapy (MIT)**-**informed approach is that many adolescents have difficulties understanding and naming what they feel or considering their negative views about themselves and others are not matter of fact, but just ideas. As a consequence, psychotherapy with adolescents is likely to benefit from focusing on their capacity to understand mental states and use this knowledge adaptively.

Several studies showed that skills related to metacognition (33, 34) and mentalizing (35) are important in helping adolescents to understand themselves and others. Mentalization is the ability to think and reflect on one’s own experiences and formulate interpretations of one’s and others’ behavior (33, 36). It involves socio-cognitive functions, including the recognition of emotions, theory of mind, mind-reading, and reflective function (37).

In adolescents with borderline (confused mentalizing) and narcissistic (excessive certainty about others’ mental states) personality traits, abnormal mentalizing was found to mediate the effects of adverse childhood experiences on their development (38).

Poor metacognition was found in various mental conditions (39–41), and significant increases in adolescents’ metacognitive abilities were observed after completing MIT (42, 43). An intervention that combines symptom-focused work with MIT (42–44) holds promise to improve treatment adherence and outcomes in adolescents with a wide array of symptoms and behavioral problems.

MIT for adults with personality disorders (PDs) has received empirical support from a series of case studies (45–47), pilot non-controlled studies (48), and randomized controlled trials (RCTs) (48). In adolescence, it has been successfully applied in early psychosis (41) and an RCT of adolescents with avoidant PD. (49) MIT combined with mentalization-based treatment improved outcomes and was associated with low dropout rates in avoidant PD. (50) Some aspects of MIT were included in a DBT-based protocol for PD requiring hospitalization; results were satisfactory (51). An RCT of group-MIT for PDs obtained large effect sizes for the MIT vs. treatment-as-usual (TAU) group on alexithymia, mastery, and self- and other-related metacognition (48). A pilot non-inferiority RCT of metacognitive and compassion treatment vs. CBT + medication for schizotypal PD yielded larger reductions in general symptomatology (η^2^ = 0.558) and larger increases in metacognition (η^2^ = 0.734) in the experimental group vs. CBT + medication (52). Overall, MIT has shown effectiveness on symptoms and social dysfunctions.

The current preliminary observational study aimed to examine treatment adherence, safety, and efficacy of MIT treatment in adolescents. To meet this goal, we compared the outcomes of an AYA group with anxiety and/or depressive disorders receiving MIT therapy with another receiving TAU (consisting of other psychotherapies not focused on metacognition but well-established in our service).

Here, we tested whether the group receiving MIT-informed psychotherapy, i.e., a combination of symptom work promoting metacognition and counteracting maladaptive interpersonal schemas was able to (a) guarantee treatment adherence as assessed with a number of dropouts; (b) be effective in terms of global psychopathology and functioning. We compared the group receiving MIT-informed therapy with TAU as routinely delivered in our unit.

## 2. Methods

### 2.1. Study design and procedure

This study was a longitudinal, prospective, naturalistic, observational study, conducted in a hospital psychiatric service dedicated to Adolescents and Young Adults (AYA). The Fondazione Policlinico Universitario A. Gemelli IRCCS ‘Early Intervention for Adolescents and Young Adults’ service provides outpatient visits and day-hospital admissions. Help-seeking patients were referred by external practitioners and other institutions. They were visited by psychiatrists of the AYA service, who assessed them in terms of diagnosis and study eligibility. Either patients or, if underage, their parents or legal tutors received adequate information regarding study aims and procedures and provided consent to participate. They were informed they could receive 16–24 weekly sessions of individual psychotherapy (but at least 8 sessions were required for being included in the study), each lasting 50 min, along with the possibility of receiving pharmacotherapy. The total duration of psychotherapy could vary from 2 to 6 months. Patients who adhered to the study received baseline assessment by their treating psychiatrists and were introduced to their psychotherapists. Treatments started within 2 weeks after assessment. The assessment carried out at baseline was repeated at the end of treatment. When this occurred earlier than the 6-month endpoint, results were carried forward for statistical purposes.

Psychotherapy could be carried out at AYA service, but patients could opt for private psychotherapy.

### 2.2. Participants

Patients referred to the service from May 2020 to March 2022 (which happened to be during the COVID-19 pandemic) were screened for eligibility. Participants had to be at least moderately proficient in Italian. Among the 111 patients screened, 60 (54%) met inclusion criteria and completed baseline and endpoint assessments. Recruitment implied assignment to MIT or TAU on the basis of patient preference and therapist availability, with both treatments being presented as potentially equivalent and no effort being made to persuade patients (or their legal tutors/parents, if patients were not of legal age) to prefer one or another. Hence, any difference that might arise in sociodemographic parameters between MIT and TAU would only be attributed to chance.

### 2.3. Exclusion criteria

Exclusion criteria were the presence of severe systemic diseases, intellectual disability or borderline functioning, psychosis, psychoactive substance use or severe eating disorders needing inpatient treatment, traumatic cranial injury, severe neurological disorders, failure to provide informed consent, and current or past psychotherapy experience. Patients were free to withdraw consent at any moment. Those withdrawing consent were instantly assessed upon withdrawal, and their results were carried forward in the analyzes. Patients who failed to initiate treatment within 2 weeks from baseline and patients/parents who refused treatment or participation in the study were excluded.

### 2.4. Assessments and outcome measures

A baseline assessment was conducted at the first visit. Endpoint assessment was set at 6 months after baseline; when assessments were made before this term, they were carried forward to the endpoint. Patients’ developmental and family histories were investigated at baseline with a semi-structured interview. Primary outcomes were the reduction of psychometric scale scores and the increase in the functioning assessment.

The *Global Assessment of Functioning (GAF) scale* (53) rates social, occupational, and psychological functioning. Scores range from 1 (“severely impaired”) to 100 (“extremely high functioning”). In this study, we used the Italian version of the scale included in the DSM-IV-TR (54), which showed good psychometric properties in Italian populations of adolescents and young adults (55, 56). Cronbach’s alpha was found to be 0.74, indicating good reliability (57).

Clinical Global Impression–Severity (CGI-S) scale (58) is a frequently used 7-point Likert scale (59). Higher scores indicate worse psychopathology. CGI-S measures the severity of patients’ illness and its improvement over treatment. Cronbach’s alpha was 0.998, which indicates excellent reliability (60).

*Brief Psychiatric Rating Scale (BPRS)* is a 7-point Likert scale (plus the option of considering an item not rated) used in order to assess the level of general psychopathology around a broad range of symptoms. A higher score indicates more severe psychopathology. It has been consistently used also as a measure of treatment change (61). The purpose of BPRS is to broaden the symptom spectrum investigated, for a psychopathological profile definition (62). Cronbach’s alpha was found to be 0.87, indicating good reliability (63).

A response was considered an at least 50% decrease from the baseline of BPRS scores, and at least a 2-point drop from baseline on the CGI-S or a CGI-S score of ≤3, while a score of 1 or 2 on the CGI-S was considered a remission.

### 2.5. Interventions

#### 2.5.1. Metacognitive interpersonal therapy

MIT aims to help patients improve their ability to understand their mental states, so they become a ground for more adaptive strategies to deal with symptoms and improve social functioning. Maladaptive interpersonal schemas encompass images of self and self-with-other and are common in PDs. They include negative core self-images (“I am unlovable” or “I have no value”) and reactions to others, e.g.: “if I express my need to be appreciated, the other person will be critical, and I will become sad, confirming my idea of having no value.” From early MIT stages, therapists help patients become aware of the schemas that guide them, gain distance from their underlying negative self-images, and promote initial access to more benevolent representations of self and others. In the manualized form applied here (43), MIT-informed treatment included a joint formulation of shared goals, helping patients recognize and pursue their needs/desires for attachment, appreciation, exploration, and group inclusion (42, 43). MIT-informed psychotherapy attempted to engage adolescents in practices such as guided imagery, chairwork, bodywork (64), and role-playing to encourage their involvement in activities, aimed at modifying their maladaptive schemata and creating new ways to derive meaning from social interactions.

Importantly, during this protocol, MIT-informed treatment included symptom-specific, empirically supported techniques, e.g., behavioral activation for depression or graded exposure to different forms of anxiety (42). One MIT-certified therapist with 1-year MIT experience treated all MIT-informed patients and received 1-h supervision fortnightly by one of the MIT developers.

#### 2.5.2. Treatment-as-usual (TAU)

The control group received TAU that consisted of individual psychotherapy delivered according to practitioners’ preferred orientation, mostly psychoanalytic/psychodynamic. Most psychotherapists conducting TAU had a psychoanalytic/psychodynamic orientation and were supervised by senior colleagues.

#### 2.5.3. Patient assignment to groups

All patients were offered integrated multidisciplinary care. Patients were assigned to MIT or TAU according to their preference and therapist availability. All patients who started psychotherapy received sufficient information about both types of therapeutic approaches and additional information about the psychotherapy they chose. In some cases, psychological therapy and medication were administered together, while in other cases, patients refrained from taking medication or they dropped out of therapy immediately and were not included in the MIT-TAU comparison. Three patients later dropped out, one from MIT and two from TAU. Psychotherapy sessions at the service’s premises or in private locations lasted 50 min each. At least eight sessions were required to include the patient in data analysis; assessments were processed through the last observation carried forward (LOCF) method.

#### 2.5.4. Other interventions

Concurrent psychiatric counseling was delivered in order to evaluate the need for medication or dose changes for some participants. Psychiatric visits (not psychotherapy sessions) were scheduled weekly for 2 weeks, then monthly for another two visits, and then bi-monthly. Visits were scheduled based on the patient’s clinical conditions and in agreement with the patient/family, but other visits could be added at patients’/tutors’ requests. Parents in both groups of patients were offered family or couple psychotherapy delivered by therapists working in the community.

### 2.6. Safety assessment

We assessed safety with spontaneous reporting of adverse events weekly. The caring physicians filled in a list of possible adverse events, especially focusing on the common side effects of medications used in this study, but also including items on suicidal thinking and attempts, self-harm ideation, and acts. These were labeled “severe,” “moderate,” or “mild.” Participants were not provided with the list to avoid being overconcerned about adverse events and being influenced in their perceptions. Safety data collection was identical for the two groups.

#### 2.6.1. Ethics statement

The study was approved by the ethics committee of Fondazione Policlinico Universitario A. Gemelli IRCCS, ID 5025 Prot. N 0020268/22 of June 14, 2022. All patients and parents were informed and signed informed consent.

### 2.7. Statistical analyses

We performed descriptive statistics to assess the sample age, sex, psychopathological diagnosis, drug therapy, and type of psychotherapeutic delivered. For the main study variables, the observed median, mean, and standard deviation were calculated. Primary outcome measures were pre–post GAF, BPRS, and CGI changes. To test sample normality of distribution, we used the Shapiro–Francia and Anderson–Darling tests that yielded *W′* = 0.977 (*p* = 0.037) and *W* 0.766 (*p* = 0.045), respectively, both ruling out normality. Hence, we turned to non-parametric tests. To analyze overall treatment response (changes in symptoms and functioning over time), we conducted non-parametric repeated-measures ANOVA. Spearman’s correlations between the scores of the 3 scales were analyzed in the two-time measures. To compare groups of patients who had undergone different types of psychotherapy, we used the repeated-measures ANOVA and *post-hoc t-test*. Statistical analysis was performed using the R 4.1 version (65). *p*-values were two-tailed; statistical significance was set at *p* < 0.05.

## 3. Results

Participants were in the age range of 13–23 years. Supplementary Figure S1 shows the flowchart of participants throughout the study. The total sample (mean age 16.7 ± 2.59) consisted of 40 female participants (66.67%) and 20 male participants (33.33%). In total, 30 participants were diagnosed with mood disorders (50%), including 13 with depressive disorders and 17 with bipolar disorders; 15 (25%) had anxiety disorders; 5 (8%) had diagnosed adjustment disorder; 4 (7%) had disruptive, impulse-control, and conduct disorders; 3 (5%) had non-underweight eating disorders; and 3 (5%) had diagnosed obsessive-compulsive disorder. Table 1 illustrates demographic characteristics, baseline tests, and between-group differences, suicidal, and self-harm symptoms.

**Table 1:** Sociodemographic characteristics of our sample subdivided according to the treatment received.

Demographics	MIT	TAU	*F*/*χ*^2^	*p*
	*N* = 18	*N* = 39		
Sex (female)	9	30	*χ*^2^ = 3.23	0.07
Dropouts	2	9	*χ*^2^ = 0.06	0.79
Age (x̄ ± SD)	*x̄* =16.75 ± 2.47	*x̄* = 16.7 ± 2.59	*F* = 0.17	0.67
*Educational level*				
Primary school	2	2	*χ*^2^ = 0.97	0.32
Middle school	9	27		
High school	7	10		
Suicidal ideation*	6	15	*χ*^2^ = 0.61	0.43
Suicide attempts*	1	8	*χ*^2^ = 2.03	0.15
Self-harm behaviors*	9	17	*χ*^2^ = 0.91	0.33

MIT, metacognitive interpersonal therapy; SD, standard deviation; TAU, treatment-as-usual; x̄, mean. *lifetime history of.

All participants were born in Italy; 27% (*n* = 16) were second-generation immigrants and had at least one parent born in a foreign country. No significant baseline differences were found regarding all variables. Spearman matrix correlations between scores on the psychometric scales (GAF, CGI, and BPRS) and a high number of emergency unit access confirmed that the instruments were capable of detecting the severity of patients’ symptoms (Table 2).

**Table 2:** Correlation matrix of clinical measures.

Measures	Spearman’s ρ	GAF BL	Emerg.Hosp.	CGI-S	BPRS BL
GAF BL	*ρ*	—			
	*p*	—			
Emerg.Hosp.	*ρ*	−0.644***	—		
	*p*	< 0.001	—		
CGI-S	*ρ*	−0.765***	0.523***	—	
	*p*	< 0.001	< 0.001	—	
BPRS BL	*ρ*	−0.633***	0.485***	0.683***	—
	*p*	< 0.001	< 0.001	< 0.001	—

***p < 0.001. BL, baseline; BPRS, Brief Psychiatric Rating Scale total scores; CGI-S, Clinical Global Impressions – Severity scale; Emerg.Hosp., emergency hospitalizations; GAF, Global Assessment of Functioning; *p*, statistical significance probability; rho (ρ), Spearman’s correlation coefficient.

Descriptive and statistical analyzes were carried out to assess whether MIT was feasible in terms of adherence and preliminary outcomes vs. TAU. We describe first the results at the level of the total sample, and then, we compare those who received MIT (*n* = 18) with those undergoing TAU (*n* = 39). Participants (*n* = 3) who refused psychotherapy were excluded from these analyzes.

As regards overall intervention adherence, 12 patients (17%) dropped out. There were 2 patients in the MIT group (11%) and nine patients (19%) in the TAU group. Concerning overall treatment response, repeated-measures ANOVA (non-parametric) was performed to assess changes in symptoms and functioning over time: GAF BL vs. GAF at the 6-month endpoint (<*χ*^2^ = 6.61, *p* < 0.001), BPRS BL vs. BPRS at the 6-month endpoint (*χ*^2^ = 6.77, *p* < 0.001), and CGI BL vs. CGI at the 6-month endpoint (*χ*^2^ = 7.20, *p* < 0.001). All differences in psychometric scores from baseline to the 6-month endpoint were significant and in the improvement direction in all groups. Scores on psychometric scales are shown in Table 3.

**Table 3:** Scores on the psychometric scales at baseline and 6-month endpoint of the entire sample (*N* = 60) and of the sample subdivided according to psychotherapy received [MIT (*n* = 18) vs. TAU (*n* = 39)].

Scales	x̄	SE	Effect size^#^	95% CI	*p*
	Entire sample				
GAF BL	6.22	0.119	−1.273	From −1.611 to −0.929	<0.001
GAF 6-mo.	7.40	0.153			
BPRS BL	51.65	1.892	1.633	From 1.242 to 2.017	<0.001
BPRS 6-mo.	33.55	1.087			
CGI-S BL	3.85	0.134	0.928	From 0.622 to 1.229	<0.001
CGI-S 6 mo.	2.33	0.152			
	MIT vs. TAU				
GAF BL MIT	6.11	0.179	0.210	From −0.352 to 0.769	0.464
GAF BL TAU	6.31	0.161			
BPRS BL MIT	54.17	2.324	−0.331	From −0.892 to 0.234	0.250
BPRS BL TAU	49.41	2.557			
CGI-S BL MIT	3.61	0.216	0.323	From −0.242 to 0.884	0.262
CGI-S BL TAU	3.95	0.176			
GAF 6 mo. MIT	7.72	0.253	−0.394	From −0.956 to 0.174	0.173
GAF 6 mo. TAU	7.26	0.197			
BPRS 6 mo. MIT	29.56	0.833	0.659	From 0.077 to 1.232	0.025
BPRS 6 mo. TAU	34.82	1.493			
CGI-S 6 mo. MIT	1.94	0.206	0.390	From −0.178 to 0.953	0.177
CGI-S 6 mo. TAU	2.56	0.204			

^#^Cohen’s d, approximately 0.2 = small, approximately 0.5 = medium, approximately 0.8, or more = large.

BL, baseline; BPRS, Brief Psychiatric Rating Scale total scores; CGI-I, Clinical Global Impressions – Improvement scale; CGI-S, Clinical Global Impressions – Severity scale; GAF, Global Assessment of Functioning; MIT, metacognitive interpersonal therapy; N, number of cases; SE, standard error; TAU, treatment-as-usual; x̄, mean; 6 mo., 6-month endpoint; 95% CI, 95 percent confidence intervals.

Of the 18 MIT patients, 4 were responders according to the at least 50% drop of BPRS scores from baseline (vs. 1 of the 38 TAU patients, *χ*^2^ = 5.765; *p* = 0.016, *χ*^2^ = 3.607 after Yates’ correction, *p* = 0.057, not significant [ns]) and 17 were responders according to the CGI-S ≥ 2-point drop from baseline or a final score of ≤3 criterion (vs. 27 of TAU patients, *χ*^2^ = 3.969; *p* = 0.046, *χ*^2^ = 2.702 after Yates’ correction, *p* = 0.100, ns). All comparisons favored MIT. According to the final CGI-S score of 1 or 2 criteria, there were 10 remitters in the MIT group vs. 21 remitters in the TAU group (*χ*^2^ = 0.0004; *p* = 0.98, ns).

In the entire group of treated AYAs, the effect size was very large for the BPRS (Cohen’s *d* = 1.515, Hedges’ *g* = 1.515) and for the CGI-S (Cohen’s *d* = 1.209, Hedges’ *g* = 1.209). For the individual groups, effect sizes were very large (“huge” according to Sawilowsky, Cohen’s *d* and Hedges’ *g* = 3.3236) for the BPRS and very large for the CGI-S (Cohen’s *d* and Hedges’ *g* = 1.518) in the MIT group, while in the TAU group, the effect sizes were large (Cohen’s *d* and Hedges’ *g* = 1.125) for the BPRS and also for the CGI-S (Cohen’s *d* and Hedges’ *g* = 1.132).

**Figure 1:**
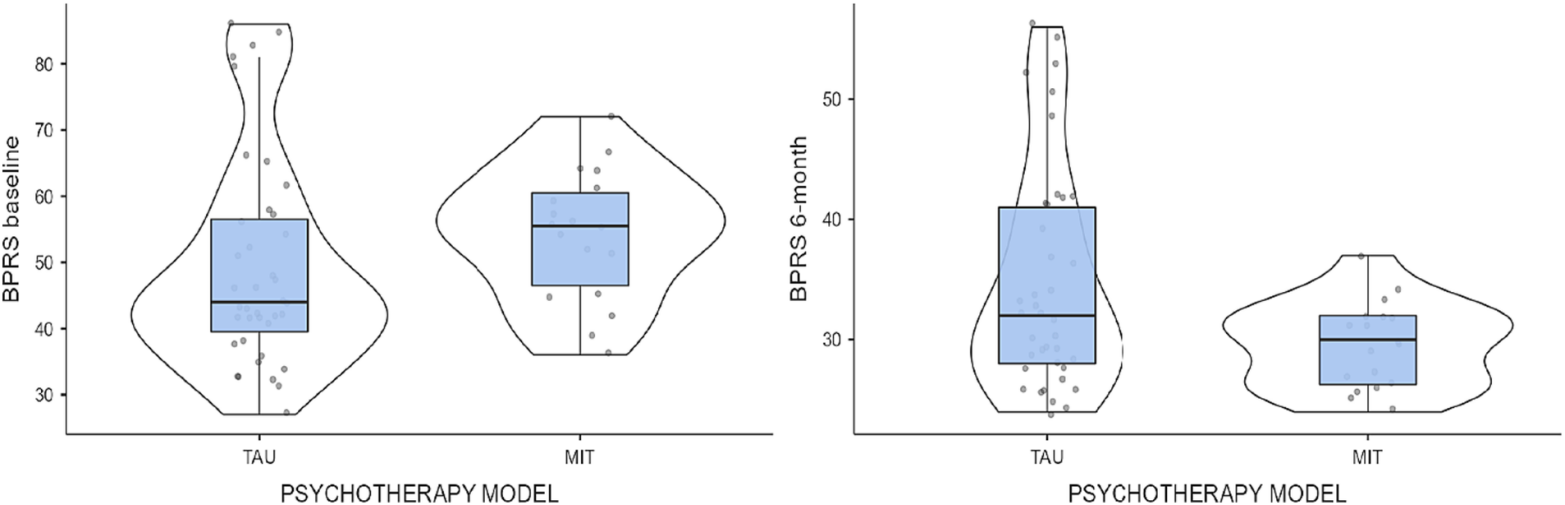
Comparison of the MIT and TAU groups as concerns the baseline–6-month comparison of BPRS scores.

As regards MIT vs. TAU, we compared outcome results on the GAF scale, BPRS, and CGI scale (BL to 6 months). On the GAF scale, the increase for the MIT group was greater than for the TAU group, *F* = 6.73, *p* = 0.01. Moreover, on the BPRS, MIT was superior to TAU, *F* = 11.8, *p* = 0.001 (Figure 1).

Concerning the CGI-S, the difference between MIT and TAU, although present, did not reach statistical significance. The *t*-test *post-hoc* analysis confirmed a significant difference between MIT and TAU after 6 months of treatment on the BPRS (*t* = 2.31; *p* < 0.05). Differences on the CGI scale did not reach significance. Separate scores by MIT and TAU groups are shown in Table 3.

### 3.1. Safety issues

Adverse events did not differ between MIT and TAU. None of the adverse events was rated “severe”; there were only three mild and transient events in the MIT group (two headaches and one gastrointestinal upset) and six in the TAU group (two headaches, two constipation, one nausea, and one vomiting). No patient developed suicidal or self-harming thinking or committed attempts or self-cutting.

## 4. Discussion

In this study, we obtained satisfactory efficacy and safety, in terms of psychometric scale drops from baseline, as well as high adherence to treatment and a low dropout rate (18.33%) in the entire group of AYA. However, MIT-informed psychotherapy was associated with a lower dropout rate than TAU (11.11% vs. 23.08%), but also lower than most dropout rates reported in literature. Both treatments proved to be efficacious, but MIT-informed psychotherapy superseded TAU on the BPRS, although not on the CGI-S.

AYA mental health services often manage symptomatologically variable and complex patients (1, 16). The importance of multidisciplinary care is now recognized in these settings (57). The present study reflects a currently growing trend in the literature to conduct studies on identifying effective psychotherapeutic interventions in AYA populations (18, 19). Treating adolescents with more effective methods is currently important, due to the general increase in global individual suffering (66). Furthermore, this population has been given particular attention after the pandemic event (10–12) due to the higher impact of the restrictions compared with other age ranges (67). The increased recognition that psychopathology in the general population received [due to a purported stress-related increased occurrence of mental disorders (68)] prompted the establishment and spread of early intervention services throughout the world (69), aiming at preventing the development of severe mental conditions or reducing the onset-detection and treatment interval. Our study carried out in a dedicated adolescent mental health service active during the pandemic involved 60 adolescents who deserved clinical attention and associated pharmacological and psychotherapeutic treatment. Obviously, psychiatric evaluation to initiate the patient to possible pharmacological (22, 23) or combined (26, 27) interventions, remains central in the psychotherapeutic intake. Overall, the study showed good efficacy of integrated care at 6-month follow-up. From this perspective, it seems interesting to explore therapeutic approaches that consider prerequisites related to knowledge of self and others’ mental states that are effective in counteracting maladaptive interpersonal patterns, which are typical of PDs (70). Among these, MIT is particularly focused on the ability to make sense of one’s own mental states and those of others. Theoretically, this approach, when provided early, could increase emotional awareness in AYAs with psychiatric symptomatology, thus allowing them to provide meaning to their emotional experience and experience and manage interpersonal relationships. This could increase the general psychological wellbeing of young people and consequently affect the reduction of anxiety-depressive psychopathology, which is very common in the AYA population (22–24). This preliminary naturalistic study showed a low dropout rate and good efficacy in patients with various psychiatric symptoms who received MIT-informed psychotherapy.

Metacognitive interpersonal therapy (MIT) (42–44) is in fact a treatment directed at increasing awareness of mental states and becoming aware of maladaptive interpersonal patterns. This treatment is applied along with empirically supported CBT techniques to promote symptom management. In our study, MIT was compared with routinely administered TAU in our unit. Consistent with previous studies (41, 47, 48, 71, 72), the results of MIT-informed psychotherapy confirmed low absolute dropout rates and higher efficacy and lower dropout compared with TAU, as well as lower dropout than other studies in the literature. Regarding efficacy, the results were good and significantly superior to TAU in terms of both symptoms and functioning; the only scale that did not reach significance was CGI.

Both treatments were safe, with only mild and transient adverse events developing in 3 patients of the MIT groups and in 5 patients of the TAU group (one patient in this group developed two symptoms). The large to very large effect sizes we observed in both groups and the entire sample indicate that help-seeking AYAs benefitted from psychotherapy.

### 4.1. Limitations

Our study confirmed previously obtained results of MIT in AYAs (41, 48, 72) but had limitations. The sample size was low and diagnostically heterogeneous to prevent us from accurately identifying and quantifying which symptoms responded to treatment. We did not assess PDs or measure metacognition using a valid scale, which constitutes the main target of MIT-informed psychotherapy. Female participants were twice as many as males. However, despite few studies reported better psychotherapy outcomes in the female sex (73, 74), others found no sex-based differences (75), and in any case, as the sex distribution in our sample did not differ significantly between MIT and TAU, it is unlikely that our results could be affected by a gender bias. Half of our sample consisted of patients with mood disorders; this probably does not reflect the proportions found in the general adolescent population (76). Furthermore, parents in both groups of patients were encouraged to consult relational psychotherapists in the community, who provided them with couple or family therapy. The progress made by parents could have affected the responses of their children to both MIT and TAU. The possible effect of the psychological adaptation of one family member on other family members’ psychological status could not be explored with this design, but it would need a design where parents of one group were exposed to relational psychotherapy and parents of another were not. This should be controlled in future studies. However, in this study, taking into account that parent psychotherapy could affect children’s outcomes and responses, these could not be affected differentially in the two groups as parents from both groups all accepted to endorse couple or family therapy. Future studies should focus on MIT-informed psychotherapy in comparison with other CBT techniques or with individual standardized psychodynamically informed psychotherapies.

In spite of the above limitations, our naturalistic study suggests that MIT is a promising treatment with the potential to help adolescents with mental health problems reduce their suffering and find their way in social life.

## Data availability statement

The raw data supporting the conclusions of this article will be made available by the authors, without undue reservation.

## Ethics statement

The studies involving humans were approved by Ethics Committee of Fondazione Policlinico A. Gemelli IRCCS, Catholic University of Sacred Heart, Rome, Italy. The studies were conducted in accordance with the local legislation and institutional requirements. Written informed consent for participation in this study was provided by the participants’ legal guardians/next of kin.

## Author contributions

EM, LM, GF, and DC conceived the study. EM, LM, GF, DJ, VZ, DV, and MS were engaged in the clinical activities of the study patient population. DJ, VZ, DV, MS, and SP directed and conducted the data collection. EM, LM, SP, and GK organized and collected the material and wrote the first draft of the manuscript. EP, GD, and RP performed literature searches. EM, LM, GF, FM, and GK wrote the Methods and decided eligibility criteria. DC, GK, RP, and GD supervised the writing of the manuscript. DC, CV, GK, and GS revised the final version of the manuscript. All authors contributed to the writing of the manuscript, read, and approved the submitted version.
