# Supplementary figures and images for: Outpatient care for adolescents’ and young adults’ mental health: promoting self- and others’ understanding through a metacognitive interpersonal therapy-informed psychological intervention
Source: Front Psychiatry. 2023 Nov 2;14:1221158. doi: 10.3389/fpsyt.2023.1221158 (PMC10651761; doi:10.3389/fpsyt.2023.1221158)

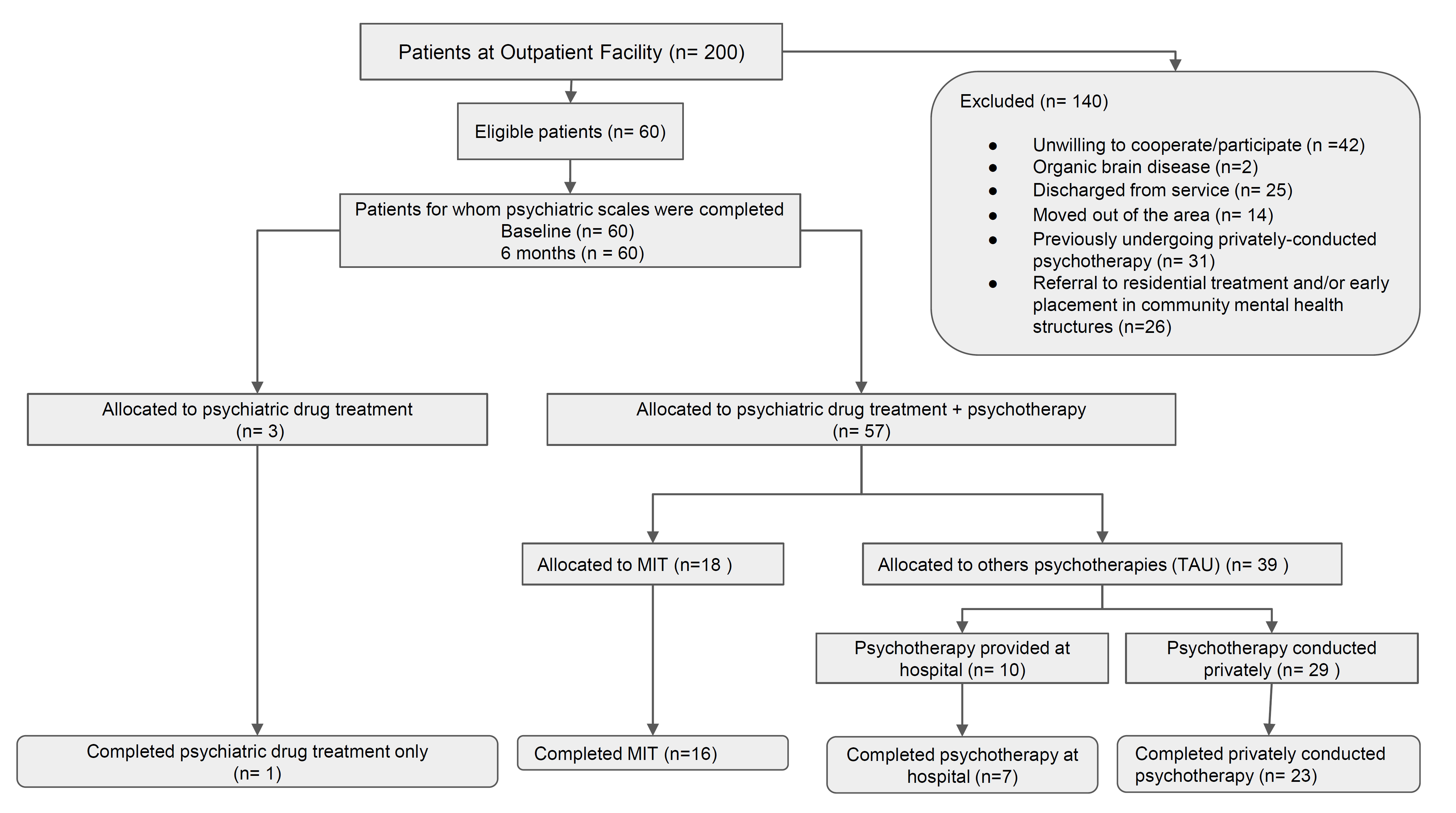

Supplement: Supplementary file 1 [file Image_1.TIFF]
